# Investigation of the Effect of the COVID-19 Pandemic Period on Respiratory Tract Viruses at Istanbul Medical Faculty Hospital, Turkey

**DOI:** 10.3390/idr16050079

**Published:** 2024-10-10

**Authors:** Sevim Mese, Aytaj Allahverdiyeva, Mustafa Onel, Hayriye Kırkoyun Uysal, Ali Agacfidan

**Affiliations:** Istanbul Medicine Faculty, Department of Virology and Basic Immunology, Istanbul University, Istanbul 34093, Turkey; draytach92@gmail.com (A.A.); onelm@istanbul.edu.tr (M.O.); hayriye.kirkoyun@istanbul.edu.tr (H.K.U.); ali.agacfidan@istanbul.edu.tr (A.A.)

**Keywords:** respiratory viruses, COVID-19 pandemic, epidemiological change

## Abstract

**Aim:** Respiratory viruses significantly impact public health, contributing to high morbidity and mortality rates in both children and adults. This study evaluates the distribution and incidence of respiratory tract viruses in our hospital from 2019 to 2022, focusing on changes post-COVID-19 pandemic. **Material and Methods:** Utilizing molecular methods, we analyzed nasopharyngeal swabs with the FTD Respiratory Pathogens 21 kit and the QIAStat Dx Respiratory Panel kit at Istanbul Faculty of Medicine. A total of 1186 viruses were detected in 2488 samples (47.6% of the total) examined with the FTD Respiratory Pathogens 21 kit between 2019 and 2022. **Results:** It was determined that the detection rates were 52.8% in 2019, 44.3% in 2020, 50.0% in 2021, and 40.0% in 2022. Notable changes in prevalence were observed for pandemic influenza A (IAV-H1N1pdm2009), parainfluenza virus (PIV)-3, rhinovirus (RV), and respiratory syncytial virus (RSV)-A/B (*p* < 0.05). RV consistently showed the highest detection rates across all years (17.6% to 7.9%). Additionally, 1276 viruses were detected in 1496 samples using the QIAStat DX kit, with 91.3% positivity in 2021 and 78.6% in 2022, highlighting the kit’s effectiveness in rapid diagnosis. **Conclusions:** This study enhances understanding of respiratory virus epidemiology during and after the pandemic, emphasizing the need for ongoing surveillance and strategic public health measures to address the evolving landscape of respiratory infections.

## 1. Introduction

Respiratory viruses pose a significant public health problem in humans by causing mild to moderate infections as well as severe lower respiratory infections (LRIs) such as pneumonia and bronchiolitis. Respiratory syncytial virus (RSV), rhinovirus (RV), influenza A virus (IAV), parainfluenza virus (PIV), and human adenovirus (hAV) are the most common acute viral LRIs. Rhinovirus (RV), previously believed to cause only mild upper respiratory tract diseases, has been found to be associated with acute and chronic LRIs, including asthma exacerbations and chronic obstructive pulmonary diseases [1,2,3]. Recently discovered respiratory viruses such as human metapneumovirus (hMPV), human bocavirus (hBoV), and human coronaviruses (hCoVs) have been associated with LRIs in children [4,5,6].

According to the WHO’s 2013 data, LRIs caused more than 2.6 million deaths worldwide, making them the fifth leading cause of death. In the WHO’s 2013 global burden of disease study, LRIs were the second leading cause of disability-adjusted life years [7].

By 2019, the data show that LRIs remained in the fourth place globally as a factor threatening human life. LRIs caused 488.9 million (95% UI: 457.6–522.6) event cases and 2.4 million (2.3–2.7) deaths worldwide in 2019. The global age-standardized incidence and death rates for LRIs in 2019 were 6295 and 34.3 per 100,000, respectively. The data indicate a 23.9% reduction in incidence and a 48.5% decline in mortality rates since 1990.

Recently, with the development of molecular techniques, detection of respiratory viruses, seasonal distributions, and monitoring of epidemics are being performed more proactively. These techniques also allow monitoring changes in the diversity of circulating respiratory viruses depending on pandemic periods. Studies have reported differences in the epidemiology of respiratory tract viruses after the COVID-19 pandemic [8,9,10,11]. These rates depend on the rates of COVID-19 detection, that differed between studies. For example, latest developments in artificial intelligence showed improved diagnostic rates when using sophisticated algorithms.

In this study, we aimed to evaluate the distribution of respiratory tract viruses diagnosed via molecular methods in our laboratory, starting from the pre-pandemic period of 2019 until the post-pandemic period of 2022. Therefore, it will provide basic findings for the discussion of changes in the prevalence of respiratory viruses in the pre-pandemic, pandemic, and post-pandemic periods, together with epidemiological characteristics. These findings will contribute to the planning of public health measures in preparation for future pandemics.

## 2. Materials and Methods

Respiratory tract virus tests performed in Istanbul University, Istanbul Faculty of Medicine Virology (IUIFM) and Basic Immunology Department Laboratory between May 2019 and June 2022 were investigated. The FTD Respiratory Pathogens 21 kit (Fast Tract Diagnostics, Esch-sur-Alzette, Luxembourg) was generally used for the detection of respiratory tract viruses in our laboratory. The QIAStat Dx Respiratory Panel kit (Qiagen GmbH, Hilden, Germany), a syndromic test, began to be used for some special cases such as intensive care, emergency pediatric, coinfection, and epidemic requiring urgent alert after the onset of the COVID-19 pandemic, as it quickly detects viruses. Although both kits are based on the multiplex real-time PCR method, during the application of this syndromic panel kit, all reagents and the nasal swab sample are placed into a QIAstat-Dx Analyzer 1.0 via a cartridge system. Then sample preparation, extraction, and RT-PCR steps are performed in a fully automatic manner without any manual intervention. Thus, it provides prompt and easy management with minimal risk of contamination for patients with serious respiratory problems [12,13].

The QIAStat Dx Respiratory Panel kit, which we use for rapid diagnosis in our laboratory and can also detect SARS-CoV-2, is applied in the Rapid Syndromic Respiratory Panel (HSSP). The FTD Respiratory Pathogens 21 kit, which we prefer for the diagnosis of more stable patients, is used in our laboratory with the Viral Respiratory Panel (VSP). With the VSP test, IAV; IAV-H1N1 pandemic 2009 virus (IAV-H1N1pdm2009); influenza B virus (IBV); parainfluenza virus (PIV)-1, -2, -3, and -4; RSV-A/B; human adenovirus (hAV); human coronavirus (hCoV) 229E; OC43; NL63 and HKU1; human bocavirus (hBoV); enterovirus (EV); human paraechovirus (hPeV); rhinovirus (RV); human metapneumovirus (hMPV); and Mycoplasma pneumonia (*M. pneumonia*) can be detected. The following can be detected using the HSSP test: IAV, IAV-H1N1pdm2009, IAV-subtype H3, IBV, hCoV229E/HKU1/NL63/OC43, severe acute respiratory syndrome coronavirus-2 (SARS-CoV-2), PIV-1/2/3/4, RSV-A/B, hMPV-A/B, hAV, hBoV, RV/EV, *M. pneumonia*, Legionella pneumophila (*L. pneumophila*), and Bordetella pertussis (*B. pertussis*).

The data of the test results were obtained from the IUIFM Information Processing System. Statistical analysis was carried out using the SPSS program (version 21, IBM Corp., Armonk, NY, USA). Pearson’s chi-square analysis was used to compare categorical data. Ethics committee approval was obtained from the Clinical Research Ethics Committee of IUIFM (date: 21 October 2022; number: 1722).

## 3. Results

The results of the VSP and HSSP tests were analyzed separately due to differences in their content, purpose, and usage periods. A negative result indicates that none of the viruses detectable by the kit was found, while detection of at least one virus is considered a positive result.

**Figure 1:**
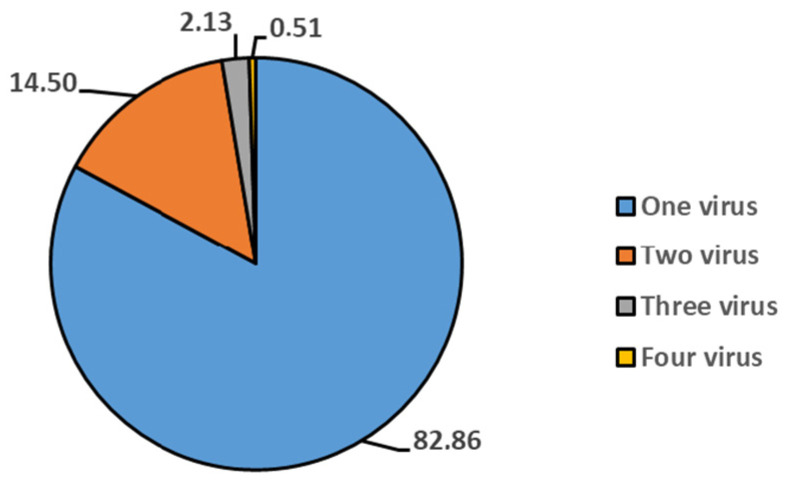
The percentage distribution of mixed viruses detected via VSP among total positive samples (*n* = 986).

VSP Test Analysis: Between May 2019 and June 2022, a total of 2488 samples were analyzed using the VSP test. Of these, 1172 samples (47.1%) were from female patients and 1316 samples (52.9%) were from male patients. The average age of patients was 21.44 years (22.19 for females and 20.72 for males). Out of the total, 1502 patients tested negative, while 986 tested positive. In the positive samples, 1186 viruses were detected: 817 samples contained one virus, 143 samples had two viruses, 21 samples had three viruses, and 5 samples had four viruses. The distribution of these viruses among the positive samples is shown in Figure 1.

Specifically, 624 (52.48%) of the detected viruses were found in males and 562 (47.26%) in females. Statistical evaluation indicated no significant differences between sexes for viruses such as PeV, hBoV, hMPV, EV, PIV-1–4, IBV, RV, hCoV-HKU, hCoV-NL63, hCoV-229E, hCoV-OC43, RSV, and M. pneumonia. However, AV (*χ*^2^ = 4.910, *p* = 0.027), IAV (*χ*^2^ = 11.810, *p* < 0.01), and IAV-H1N1pdm09 (*χ*^2^ = 8.098, *p* < 0.01) showed significant sex-based distribution differences (Table 1).

The distribution of respiratory tract viruses was analyzed across three age groups: 0–18, 19–50, and over 50. Significant differences were found, except for hCoV NL63/229E/HKU, EV, IAV-H1N1pdm09, hMPV, and PIV-1 (Table 2).

**Figure 2:**
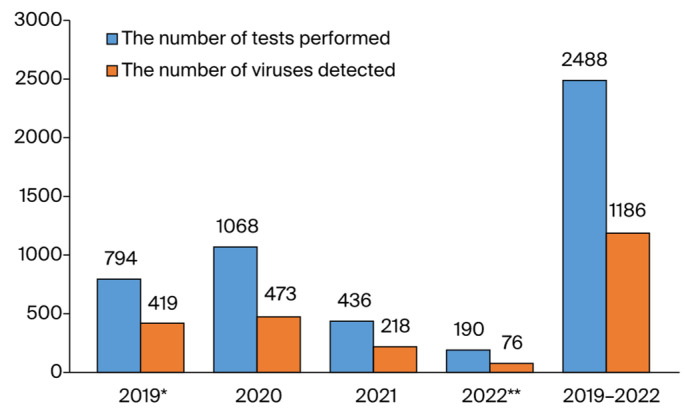
A visual representation of the distribution of respiratory viruses detected via the VSP test according to year. * The data of 2019 stars from May. ** The data of 2022 ends on May.

Overall, 1186 viruses were identified in 2488 samples (47.6% positivity) with the FTD Respiratory Pathogens 21 kit, revealing detection rates of 52.8% (419/794) in 2019, 44.3% (473/1068) in 2020, 50.0% (218/436) in 2021, and 40.0% (76/190) in 2022 (Figure 2).

**Figure 3:**
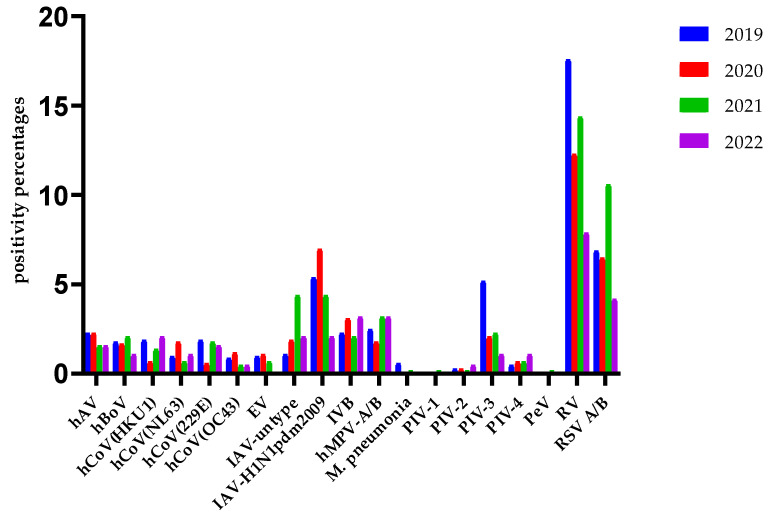
The distribution of the positivity rates of the VSP test in the years 2019–2022. hAV: human adenovirus; hBoV: human bocavirus; hCoV: human coronavirus; EV: enterovirus; IAV: influenza A virus; IVB: influenza B virus; hMPV: human metapneumovirus; *M. pneumonia*: *Mycoplasma pneumonia*; PIV: parainfluenza virus; PeV: paraechovirus; RV: rhinovirus; RSV: respiratory syncytial virus.

Notable detections in 2019 included hAV (18 cases), hBoV (14), hCoV (HKU1: 15, NL63: 8, 229E: 15, OC43: 7), EV (8), IAV (9), IAV-H1N1pdm2009 (43), M. pneumonia (5), hMPV (20), PIV (1: 2, 2: 0, 3: 41, 4: 4), IBV (18), RV (137), and RSV-A/B (55). The detections in subsequent years varied, with significant differences noted across years for each virus (Figure 3).

**Figure 4:**
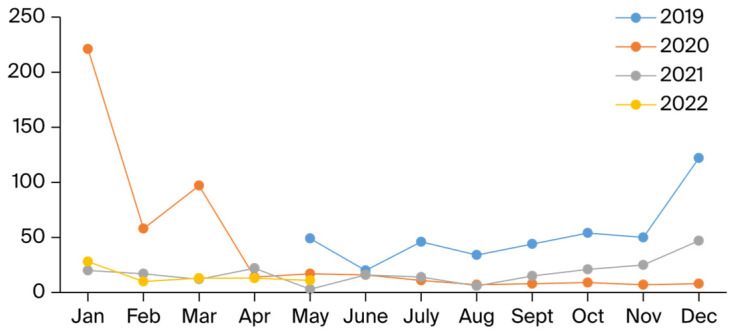
The positivity of the VSP test by months within the period May 2019–May 2022.

Seasonal distribution analysis revealed that the VSP test results differed significantly by season for rhinovirus (*χ*^2^ = 9.525, *p* = 0.023) and influenza AH1N1 (*χ*^2^ = 9.599, *p* = 0.022), as illustrated in Figure 4.

**Figure 5:**
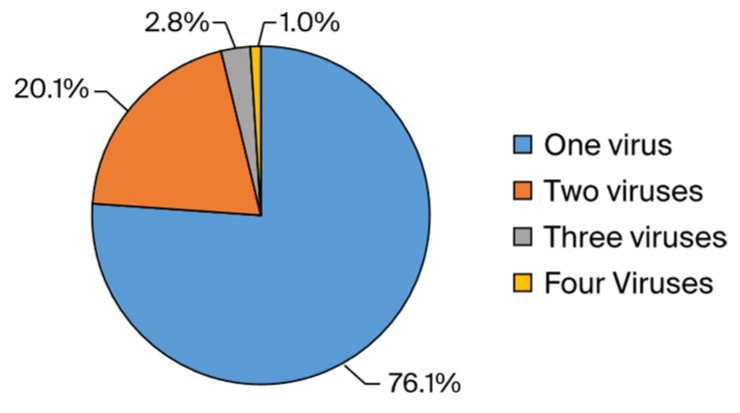
Illustrates the percentage distribution of mixed viruses detected via HSSP in positive samples (*n* = 991).

A total of 1496 samples were analyzed via HSSP test between 2021 and 2022. Of these, 631 samples (42.18%) were from female patients and 865 (57.82%) were from male patients. The average age of all patients who underwent HSSP testing was determined to be 6 years old (6 for female patients, 5 for male patients). Among the total, 505 patients tested negative, and 991 exhibited positive results. A total of 1276 respiratory viruses were identified, including 754 samples with a single virus, 199 samples with two viruses, 28 with three viruses, and 10 with four viruses. The distribution of mixed viruses in the total positive samples is illustrated in Figure 5.

HSSP Test Analysis: Between 2021 and 2022, a total of 1496 samples were analyzed via the HSSP test, comprising 631 (42.18%) from females and 865 (57.82%) from males. The average age of patients was 6 years (6 for females, 5 for males). Of these, 505 tested negative, while 991 were positive, identifying a total of 1276 respiratory viruses. This included 754 samples with a single virus, 199 with two viruses, 28 with three, and 10 with four. The distribution of mixed viruses among positive samples is shown in Figure 5.

A total of 1276 viruses were identified via the HSSP test, with 736 (57.7%) in males and 540 (42.3%) in females. Cross-tabulation analysis showed no significant differences for several viruses, but PIV-4 indicated a significant gender difference (*p* = 0.038) (Table 3).

Table 4 presents the distribution of respiratory tract viruses detected via the HSSP test according to three age groups—0–5, 6–25, and over 25. A significant difference was observed in the distribution of respiratory tract viruses, with the exception of hCoV-OC43/HKU-1, IBV, hMPV, PIV-1, -2, -4, and SARS-CoV-2.

**Figure 6:**
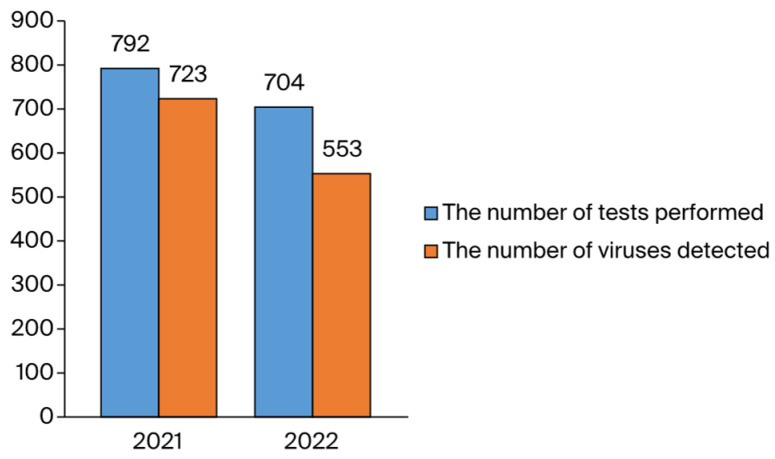
A graphical representation of the distribution of respiratory viruses detected via the HSSP test in the examined years.

The HSSP test determined a positivity rate of 91.3% (723/792) in 2021 and 85.7% (1.276/1.496) in 2022, while the overall positivity rate across all years was found to be 85.3% (1.276/1.486) (Figure 6).

**Figure 7:**
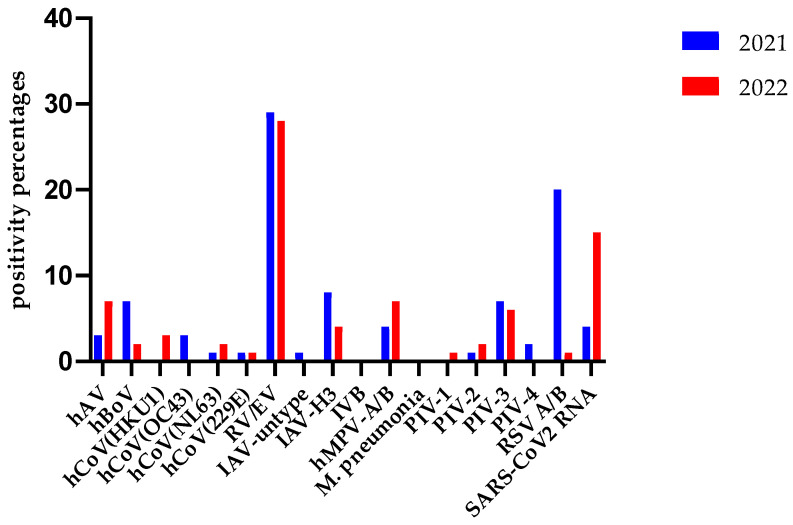
The distribution of the positivity rates of the viruses included in the HSSP test in 2021 and 2022. hAV: human adenovirus; hBoV: human bocavirus; hCoV: human coronavirus; RV/EV: entero-virus/rhinovirus; IAV: influenza A virus; IVB; influenza B virus; hMPV: human metapneumovirus; PIV: parainfluenza virus; RSV: respiratory syncytial virus, SARS-CoV-2: severe acute respiratory syndrome coronavirus-2.

In 2021, detected viruses included hAV (22 cases), hBoV (57 cases), hCoV HKU1/NL63/OC43/229E (2, 6, 20, and 10 cases, respectively), RV/EV (231 cases), IAV-untyped (4 cases), IAV-H3N1 (67 cases), hMPV (34 cases), PIV (0, 8, 53, and 19), IBV (0), RSV A/B (155 cases), and SARS-CoV-2 (35 cases). The numbers of respiratory viruses detected in the HSSP test in 2022 were as follows: 49, 13, 22, 11, 0, 5, 199, 0, 28, 49, 5, 14, 39, 1, 3, 10, and 105, respectively (Figure 7).

**Figure 8:**
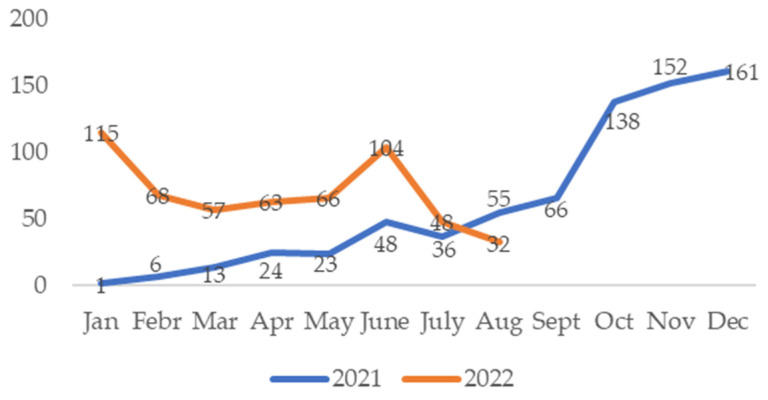
The positivity of the HSSP test each month from January 2021 to August 2022.

A cross-tabulation of the HSSP test results of the participants according to the seasons revealed that the following viruses exhibited different distribution according to the season: RV/EV (*x*^2^ = 24.790; *p* = 0.00; Pearson chi-square), RSV-A/B (*x*^2^ = 258.954; *p* = 0.000; Pearson chi-square), PIV-4 (*x*^2^ = 43.679; *p* = 0.000; Fisher’s exact test), PIV-1 (*x*^2^ = 7.155; *p* = 0.000; Fisher’s exact test), hCoV-HKU1 (*x*^2^ = 25.505; *p* = 0.000; Fisher’s exact test), hCoV-229E (x^2^ = 8.605; *p* = 0.024; Fisher’s exact test), and SARS-CoV-2 *(x*^2^ = 48.677; *p* = 0.00; Pearson chi-square). The course of HSSP test positivity each month in 2021 and 2022 is presented in the accompanying figure, Figure 8.

## 4. Discussion

Respiratory virus infections, especially LRIs, cause high mortality and morbidity rates in children and adults worldwide [12]. Approximately 4 million people die due to acute respiratory infections every year, and 98% of these deaths are caused by lower respiratory tract infections. High mortality rates occur in low- and middle-income countries, particularly in infants, children, and the elderly. One of the most common reasons for consultation or admission to healthcare institutions in pediatric services is acute respiratory infections [14].

It is known that at the time when SARS-CoV-2 first emerged from China, many seasonal viruses were circulating in the Northern Hemisphere, including IAV and IBV, RSV, hMPV, RV, PIV-1–4, and other hCoVs [15]. Studies from different parts of the world have shown that there have been changes in the epidemiological features of respiratory tract viruses since the onset of the COVID-19 pandemic [8,16,17,18,19]. For this purpose, we analyzed the data of respiratory tract viruses detected via molecular methods in our laboratory and revealed findings that reflect the demographic characteristics of respiratory tract infections in the pre-pandemic and post-pandemic periods. The first COVID-19 case in Turkey was seen on March 10, 2020, a day before the World Health Organization declared a pandemic. However, the pandemic had an intense impact on Turkey in 2021 [20,21]. Moreover, prolonged viral positivity has been documented in cases of substantial SARS-CoV-2 reinfection [22]. In our study, 2019 and 2022 were evaluated for the pre-pandemic and post-pandemic periods, respectively. Therefore, we evaluated the years 2019 and 2022 in our study as pre-pandemic and post-pandemic periods, respectively.

In order to compare the COVID-19 pandemic and pre-pandemic period in the emergency department (ED) visits made within the scope of the National Syndromic Surveillance in the USA, the 773,74,84 ED visits of the pandemic period (weeks 10 to 52 of 2020) and the 252,826,677 ED visits of the pre-pandemic period (2017, 2018, and weeks 10 to 52 of 2019) were evaluated. As a result of this study, the percentage positivity of influenza viruses, RSV, PIV, hAV, and hMPV was found to be lower in 2020 compared to 2019. Although the testing volume has decreased, the percent positivity for RV/EV has been reported to be higher in the last weeks of 2020 compared to 2019 [16]. In our study, while influenza A was detected at a rate of 5.4% with the VSP test in 2019, it was detected at a rate of 4.4% in 2021, when the pandemic was active. Since the VSP test does not detect SARS-CoV-2, samples from cases with negative routine SARS-CoV-2 PCR test results were sent to our laboratory by the clinics for VSP testing. Therefore, since an evaluation was made among the non-SARS-CoV-2 viruses detected by the VSP test, there was no tremendous decrease in their rates.

In a study investigating three periods in Italy (May 2018–April 2019, May 2019–April 2020, and May 2020–April 2021), the positivity rates of respiratory viruses other than SARS-CoV-2 were 1094/2198 (49.8%), 1251/3211 (39%), and 107/797 (13.4%), respectively. The results of this study also support the hypothesis that the pandemic changed the typical seasonal trend in respiratory viruses. Flu viruses (IVA/B) or RSV, which are typically winter viruses, were not detected. From 2020–2021, among respiratory viruses, the most common pathogen was RV, followed by bocavirus and seasonal hCoV, among the non-SARS-CoV-2 viruses [17]. It was determined that the incidence of RV/EV in children with acute respiratory illness in the USA, which decreased during the initial period of the pandemic, returned to pre-pandemic levels after October 2020 [23]. In our study, it was revealed that the incidence of RV/EV was the least affected by the pandemic compared to other respiratory viruses (Figure 3 and Figure 7).

In a study conducted in children in Moscow, non-SARS-CoV-2 cases were compared between 2018 and 2020. Accordingly, the detection rate of non-SARS-CoV-2 viruses in 2020 was 16.9% versus 37.6%, which was lower than in 2018. When non-SARS-CoV-2 respiratory viruses were compared individually in the same periods, the detection rates of RV, hAV, PIV-3, and hBoV were found to be significantly higher in 2018 compared to 2020. However, the hMPV detection rate was higher in 2020 (chi-square test, all *p* < 0.05). This study observed an increase in the median age of children with respiratory viruses during the pandemic (3 years versus 1 year). In addition, no significant difference was found in the frequency of admission to the intensive care unit (ICU) in children with SARS-CoV-2 and other respiratory tract viruses (2.7% vs. 2.9%) [18].

The analysis of non-SARS-CoV-2 viruses according to age in our study; RSV, hAV, hBoV, and PIV-3 showed significant differences between ages for both VSP and HSSP. While hCoV-OC43, IVB, PIV-4 showed a significant difference between ages in the VSP test, hCoV-229E/NL63, RV/EV, IAV-H3 showed a significant difference between ages in the HSSP test. Unlike the VSP test, since the HSSP test is applied to intensive care and emergency pediatric patient groups, age analysis results may not match exactly. Additionally, since the average age of patients undergoing the HSSP test was lower than for the VSP test (6 vs. 21.44), we analyzed different age groups (0–5, 6–25, 25+ for HSSP test; 0–18, 19–50, 50+ for VSP test).

The effect of the COVID-19 pandemic on respiratory tract viruses in children has also been studied in the Southern Hemisphere. In a comprehensive study conducted in Melbourne, 4636 respiratory virus test results (range 3758–5348) from between 2015 and 2019 (weeks 1 to 47) were compared with 3659 test results from weeks 1 to 47 of 2020, retrospectively. There was a 21% decrease in the number of tests during the pandemic period. In this study, the positivity rates of IAV, IBV, RSV, and PIV were significantly reduced in period 2 compared to period 1: 77.3, 89.4, 68.6, and 66.9% reductions, respectively (all *p* < 0.001). From week 12–47, 2020, 28,893 SARS-CoV-2 tests were performed with a 0.64% positivity rate. Influenza viruses were not detected after week 17, RSV was not detected after week 35 [19].

Agca et al. investigated changes in the epidemiology of influenza and other respiratory tract viruses during the COVID-19 pandemic period in our country. For this purpose, the researchers conducted syndromic analysis for IAV, IBV, RV/EV PIV-1–4, CoVs, hMPV, hBoV, RSV, and AV on 319 nasopharyngeal swab samples between 1 March 2020 and 28 February 2021. A total of 101 (31.7%) samples were positive, including a single virus in 88 of the nasopharyngeal samples and more than one virus in 13 samples. In this study, RV/EV was the most common virus detected in all age groups, particularly in the group of subjects of 2–5 years of age. The researchers reported that the influenza positivity rate decreased from 17.3% to 2.3% in the first year of the pandemic compared to the previous year, and hMPV infection activity did not change during the pandemic [8].

In our laboratory, 2488 VSP test results in the years 2019–2022 and 1496 HSSP test results in the years 2021–2022 were evaluated annually. In 2019, the year just before the pandemic, the overall positivity rate with the VSP test was 52.8%, while RV, RSV, and IVA were the three viruses with the highest incidence, with 17.6%, 6.9%, and 5.4%, respectively. Since the first COVID-19 case in our country was reported on 11 March 2020, there was no significant change in the general positivity rate of seasonal respiratory viruses in 2020 (44.3%) compared to 2019, due to the high positivity rates from January–March. However, it is clear from Figure 4 that the number of respiratory viruses detected by the VSP test decreased significantly after March 2020 and this continued in 2020. According to the data in Figure 3, it appears that the virus least affected by the pandemic in circulation is RV, with an incidence of 12.3%. We can explain RSV’s second place with a rate of 6.5% by the fact that the pediatric clinic in our hospital acts sensitively in terms of respiratory tract infections and is the clinic that sends the most samples.

In our study, 76.1% of the total positive results were detected as a single virus, 20.1% as two viruses, 2.8% as three viruses, and 0.1% as four viruses. In order to exclude false coinfections due to prolonged viral shedding, recurrent positive results that did not meet the reinfection criteria were excluded. Thus, it was possible to work with a clean data set in which long-term viral shedding affecting the positivity rates was excluded. However, the quadruple coinfection rate of 0.1% can be attributed to the sensitivity and specificity of the kit used.

While the COVID-19 pandemic continued in full force in 2021, the positivity rate of seasonal viruses with the VSP test was 50%. The virus which managed to maintain its effectiveness during the pandemic in 2021 was RV, with a rate of 14.4%. For the HSSP test, which we added to our test scale for specific conditions in 2021, the highest incidence rates were found to be 29% for RV, 20% for RSV, and 8% for IAV-H3. RSV was detected at a high rate because the syndromic test (HSSP), which is indicated in specific situations, such as intensive care and emergency, is especially preferred by the pediatric clinic, within the framework of conscious test indication, in our hospital. This syndromic test also includes the SARS-CoV-2 virus. However, since it is mostly applied to patients who tested negative with the routine SARS-CoV-2 PCR test, we detected SARS-CoV-2 at a rate of 4% with this test.

According to the VSP test results we evaluated between May 2019 and June 2022, we determined that respiratory viruses started to increase in September 2019 and peaked in December. On the other hand, we see that the peak level of respiratory viruses continued in January 2020 and that other respiratory viruses were almost at basal levels with the declaration of the pandemic in our country in March 2020 (Figure 4). We obtained similar findings in the monthly distribution of HSSP test results, which we evaluated only in the post-COVID-19 pandemic period. The increase in respiratory viruses started in September 2021 and reached a peak level in December. In 2022 started with a lower peak level, it continued at a steady rate at serious levels between February and May and reached a peak at the same level as January in June. Although we cannot fully evaluate the winter months in 2022 due to the shortage of kits in our laboratory, our data show that respiratory viruses were detected as causative agents in a longer period after the pandemic. It has been discussed in various studies that the COVID-19 pandemic caused a change in the seasonality of respiratory viruses. In a global study, it was determined that the typical seasonal patterns of influenza and RSV changed in the post-COVID-19 pandemic period and showed earlier peaks in temperate regions. It was also determined that the duration of the influenza epidemic extended by 2.2 weeks and there was a similar increase in RSV [24]. In their study, Maison et al. observed that the virus spectrum increased in the period following the COVID-19 pandemic and that there was a four-fold increase in pediatric respiratory tract infections with a predominance in RV/EV infections [25].

In 2022, for which we can only evaluate the first half of the year, the positivity rates were determined as 40.0% with VSP and 78.6% with the HSSP test (Figure 2 and Figure 6). Even though there was a decrease in total positivity in the first half of 2022, there was an increase in virus diversity compared to 2020 and 2021, when the effects of the pandemic were intense in our country. It can be observed that there was a significant decrease in the incidence of other respiratory viruses detected via VSP for 2020 and 2021. Since we began using the HSSP test in 2021, we have not had the opportunity to compare the results of this test with those of pre-pandemic testing. However, when we evaluated the HSSP test with the VSP test for the years 2021 and 2022, the detection rates of some significant respiratory tract viruses were found to be higher. Since the tests are not applied to the same patients, high rates of HSSP do not indicate that the detection power of HSSP is higher than that of VSP. These high rates can be attributed to the application of the HSSP test to a more specific range of patients. In 2022, RV also showed the highest incidence with both VSP and HSSP testing.

## 5. Conclusions

In our study, the incidence of respiratory viruses varied by year. As a result of the analysis of the data of the VSP test between 2019 and 2022, a significant difference was obtained for the incidence of IAV, PIV-3, RV, RSV A/B. Analysis of data of the HSSP test from 2021–2022 showed that there was a significant difference in the incidence of RSV-A/B, PIV-4, PIV-3, PIV-1, hAV, hBoV, hCoV-HKU1/OC43, IAV, hMPV, and SARS-CoV-2 viruses. According to the data of both tests, while non-SARS-CoV-2 viruses were detected at much lower rates during the pandemic period, RV was the virus least affected by the pandemic. The fact that RSV ranks second can be attributed to the fact that the number of pediatric samples examined is much higher due to the awareness of the pediatric clinic in our hospital about respiratory tract infections. Conscious application of tests for the etiology of respiratory tract infections is important for effective patient management and collection of epidemiological data.

In this retrospective study, we could not examine a wide period of time before and after the pandemic due to the capacity of the hospital information technology system and its practical limitations. In order to obtain strong epidemiological data, the necessity of comprehensive and secure data access opportunities is inevitable in today’s conditions.

In our study, we also obtained data that showed that respiratory viruses differ according to age and gender. In particular, RSV, hAV, hBoV, and PIV-3 showed significant differences between ages in both the VSP and HSSP tests. According to VSP test results, hAV and IAVs differed according to gender. According to the HSSP test results, PIV-4 differed according to gender. However, it is well-known that the infection rate with respiratory viruses depends strongly on the immune state and other patient-related variables, for example, on cortisol levels [26].

As a result, it is important to monitor the variable epidemiological features of respiratory tract viruses, within the framework of changing global conditions, to determine prevention and treatment strategies for respiratory tract infections. For this reason, we believe that our study, which includes the pre-pandemic and post-pandemic periods, will be an important data source showing the effects of the pandemic for science and health authorities both in our country and worldwide. These data allow the evaluation of the effects of public health measures, host behavior, and viral factors on outbreaks, contributing to the planning of preparation stages for future pandemics.

## Figures and Tables

**Table 1:** Evaluation of respiratory viruses detected via VSP test between 2019 and 2022 according to sex.

Virus	Female	Male	*p* (Fisher’s Exact Test)
*M. pneumonia*	1	6	0.083 *
PeV	2	0	0.222 *
hAV	17	36	0.027
hBoV	17	26	0.396 **
hMPV-A/B	26	33	0.636
EV	11	12	1.000 **
PIV-1	4	3	302 *
PIV-3	37	38	0.695
PIV-2	1	0	0.720 *
IVB	27	39	0.307
PIV-4	7	9	0.992 **
RV	155	181	0.781
hCoV-HKU	16	16	0.879 **
hCoV-NL63	20	12	0.115 **
hCoV-229E	15	17	1.000 **
hCoV-OC43	10	13	0.888 **
RSV	78	100	0.362
IAV-untyped	31	21	0.001
IAV-H1N1pdm09	87	62	0.004

* Fisher’s exact test; ** continuity correction. hAV: human adenovirus; hBoV: human bocavirus; hCoV: human coronavirus; EV: enterovirus; IAV: influenza A virus; IVB: influenza B virus; hMPV: human metapneumovirus; *M. pneumonia*: *Mycoplasma pneumonia*; PIV: parainfluenza virus; PeV: paraechovirus; RV: rhinovirus; RSV: respiratory syncytial virus.

**Table 2:** The distribution of respiratory viruses detected via the VSP test according to age groups.

	0–18	19–50	50+	*p*
hAV	48	4	1	0.000
hBoV	39	2	2	0.002
hCoV-NL63	17	9	6	0.142 *
hCoV-229E	22	3	7	0.675 *
hCoV-OC43	8	5	10	0.003 *
hCoV-HKU1-	21	6	5	>0.05 *
EV	19	3	1	0.159 *
IAV-untyped	21	16	15	0.000
IAV-H1N1pdm2009	100	23	26	0.889
IVB	54	7	5	0.015
hMPV-A/B	46	4	9	0.089
*M. pneumoniae*	6	1	0	
PIV-1	5	1	1	>0.05 *
PIV-2	1			
PIV-3	65	4	6	0.000
PIV-4	15	1	0	0.039 *
PeV	2			
RV	282	27	27	0.00
RSV-A/B	152	8	18	0.00

* Fisher’s exact test. hAV: human adenovirus; hBoV: human bocavirus; hCoV: human coronavirus; EV: enterovirus; IAV: influenza A virus; IVB: influenza B virus; hMPV: human metapneumovirus; *M. pneumonia*: *Mycoplasma pneumonia*; PIV: parainfluenza virus; PeV: paraechovirus; RV: rhinovirus; RSV: respiratory syncytial virus.

**Table 3:** Evaluation of viruses that were identified as positive via HSSP, stratified by gender.

Virus	Female	Male	*p*
hAV	31	40	0.778
hBoV	29	41	0.914
hCoV-NL63	7	10	1.000 **
hCoV-229E	9	6	0.249 **
hCoV-OC43	7	13	0.678 **
hCoV-HKU1	11	13	0.865 **
EV/RV	180	250	0.818
IAV-H3	40	55	0.991
IAV-untyped	2	2	0.640
IVB	1	2	1.000 *
hMPV-A/B	37	46	0.631
PIV-1	0	5	0.65 *
PIV-2	10	12	0.914 **
PIV-3	38	54	0.881
PIV-4	13	7	0.038 **
RSV	62	103	0.205
SARS-CoV-2	63	77	0.506

* Fisher’s exact test; ** continuity correction. hAV: human adenovirus; hBoV: human bocavirus; hCoV: human coronavirus; EV: enterovirus; IAV: influenza A virus; IVB: influenza B virus; hMPV: human metapneumovirus; *M. pneumonia*: *Mycoplasma pneumonia*; PIV: parainfluenza virus; RV: rhinovirus; RSV: respiratory syncytial virus.

**Table 4:** The distribution of respiratory viruses detected via the HSSP test according to age groups.

	0–5	6–25	25+	*p*
hAV	62	8	1	0.002
hBoV	64	6	0	0.000
hCoV (229E)	8	2	5	0.00 *
hCoV(OC43)	14	5	1	0.880
hCoV(NL63)	17	0	0	0.011 *
hCoV(HKU1)	18	6	0	0.569
RV-EV	331	90	9	0.000
IAV-untyped	2	2	0	0.000
IAV-H3	47	42	6	0.000
IVB	1	2	0	0.290 *
hMPV A/B	66	15	2	>0.05
PIV-1	5	0	0	>0.05 *
PIV-2	16	5	1	0.806
PIV-3	81	10	1	0.000
PIV-4	15	5	0	>0.05
SARS-CoV-2	88	44	8/	0.186
RSV A/B	147	18	0	0.000

* Fisher’s exact test; hAV: human adenovirus; hBoV: human bocavirus; hCoV: human coronavirus; RV/EV: enterovirus/rhinovirus; IAV: influenza A virus; IVB; influenza B virus; hMPV: human metapneumovirus; PIV: parainfluenza virus; RSV: respiratory syncytial virus, SARS-CoV-2: severe acute respiratory syndrome coronavirus-2.

## Data Availability

The primary research data that support the findings of this study were obtained from Istanbul University, Istanbul Medicine Faculty Information Processing System.
